# Preference of musculoskeletal pain treatment in middle-aged and elderly chinese people: a machine learning analysis of the China health and retirement longitudinal study

**DOI:** 10.1186/s12891-023-06665-7

**Published:** 2023-06-29

**Authors:** Fengyao Mei, Shengjie Dong, Jiaojiao Li, Dan Xing, Jianhao Lin

**Affiliations:** 1 Arthritis Clinic and Research Center, Peking University People’s Hospital, Beijing, 100044 China; 2 Arthritis Institute, Peking University, Beijing, China; 3 Department of the Joint and Bone Surgery, Yantaishan Hospital, Binzhou, P.R. China; 4 School of Biomedical Engineering, Faculty of Engineering and IT, University of Technology Sydney, Ultimo, NSW 2007 Australia

**Keywords:** Musculoskeletal pain, Treatment preference, CHARLS, Machine learning

## Abstract

**Background:**

Musculoskeletal pain is a major cause of physical disability, associated with huge socioeconomic burden. Patient preference for treatment is an important factor contributing to the choice of treatment strategies. However, effective measurements for evaluating the ongoing management of musculoskeletal pain are lacking. To help improve clinical decision making, it’s important to estimate the current state of musculoskeletal pain management and analyze the contribution of patient treatment preference.

**Methods:**

A nationally representative sample for the Chinese population was derived from the China Health and Retirement Longitudinal Study (CHARLS). Information on the patients’ demographic characteristics, socioeconomic status, other health-related behavior, as well as history on musculoskeletal pain and treatment data were obtained. The data was used to estimate the status of musculoskeletal pain treatment in China in the year 2018. Univariate analysis and multivariate analysis were used to find the effect factors of treatment preference. XGBoost model and Shapley Additive exPlanations (SHAP) method were performed to analyze the contribution of each variable to different treatment preferences.

**Results:**

Among 18,814 respondents, 10,346 respondents suffered from musculoskeletal pain. Approximately 50% of musculoskeletal pain patients preferred modern medicine, while about 20% chose traditional Chinese medicine and another 15% chose acupuncture or massage therapy. Differing preferences for musculoskeletal pain treatment was related to the respondents’ gender, age, place of residence, education level, insurance status, and health-related behavior such as smoking and drinking. Compared with upper or lower limb pain, neck pain and lower back pain were more likely to make respondents choose massage therapy (P < 0.05). A greater number of pain sites was associated with an increasing preference for respondents to seek medical care for musculoskeletal pain (P < 0.05), while different pain sites did not affect treatment preference.

**Conclusion:**

Factors including gender, age, socioeconomic status, and health-related behavior may have potential effects on people’ s choice of treatment for musculoskeletal pain. The information derived from this study may be useful for helping to inform clinical decisions for orthopedic surgeons when devising treatment strategies for musculoskeletal pain.

**Supplementary Information:**

The online version contains supplementary material available at 10.1186/s12891-023-06665-7.

## Introduction

Pain is one of the most frequent reasons for patients seeking medical care. Musculoskeletal disorders are a major contributor to pain, accounting for approximately half of the final diagnosis made in patients suffering from pain [1, 2]. Musculoskeletal disorders encompass a diverse group of diseases affecting the bones, joints, ligaments and tendons, and associated soft tissues, and include more than 150 different diagnoses. Musculoskeletal pain is the most commonly presented symptom for patients with musculoskeletal disorders [3]. Among patients with chronic pain conditions worldwide, musculoskeletal pain accounted for the largest proportion of cases in all geographical regions and at all age groups [4, 5]. Musculoskeletal pain was also one of the highest causes of physical disability in 2017, with neck pain and lower back pain respectively ranking 9th and 13th among all causes ranked by disability-adjusted life years (DALYs) [6, 7]. Due to the chronic and persistent nature of musculoskeletal pain, it is associated with a huge socioeconomic burden on both patients and the healthcare system, including a bill of 213 billion dollars on the U.S. healthcare system for musculoskeletal pain management in 2011 [8].

Musculoskeletal pain may arise due to different types or a combination of musculoskeletal disorders, such as inflammation and neuropathy, and often also involves different sites, most commonly the neck, lower back, hip, and knee, and for these reasons are treated in a variety of ways [9]. However, major musculoskeletal disorders such as arthritis and bone diseases, which lead to the greatest impacts on patients and healthcare systems, have no effective treatment and require ongoing management [10]. Despite a variety of options, the therapeutic management of musculoskeletal pain remains a significant clinical challenge. Current strategies used for musculoskeletal pain management include non-pharmacological treatments (such as patient education and self-management, exercise therapy, and massage therapy), complementary therapies (such as acupuncture), and pharmacological interventions (such as non-steroidal anti-inflammatory drugs (NSAIDs)) [11]. Within the Chinese population, traditional herbal medicine may also be a strong preference for musculoskeletal pain management in both clinicians and patients [12].

Our recent national survey on the preference of orthopedic practitioners in clinical management of musculoskeletal pain revealed that the level and type of hospital, as well as the practitioner’s level of education may influence their preferences when selection treatment strategies [3]. However, information regarding the Chinese population on factors influencing patient preferences when seeking medical care for musculoskeletal pain, as well as the current status of treatment in the population are currently lacking. In this study, we used data collected from the China Health and Retirement Longitudinal Study (CHARLS), comprising a nationally distributed random sample of the Chinese population. Using the latest nationwide representative sample of the follow-up survey on health and pension, we estimated the current status of treatment for musculoskeletal pain among Chinese residents age 45 years or older in the year 2018. The results of our study indicated that patient-related factors may influence their treatment preferences for musculoskeletal pain.

## Methods

### Study population

CHARLS is a nationally representative longitudinal survey of the middle-aged and elderly population in China. The study interviewed Chinese residents aged 45 years or older and their spouses in their household, assessing their social, economic, and health status. All participants provided informed consent, and the protocol was approved by the Ethical Review Committee of Peking University (approval number: IRB00001052-11,015). A detailed description of the CHARLS has been published previously [13].

In 2008, CHARLS performed a preliminary survey in Zhejiang and Gansu provinces, respectively representing the typical conditions of east and west China. The national baseline survey was performed in 2011, and interviews were conducted in 2011, 2013, 2015 and 2018 in 150 counties and 450 communities (villages) across 30 provinces (autonomous regions and municipalities directly under the Central government). By the time the nationwide follow-up survey was completed in 2018, the study sample had covered 19,000 respondents from a total of 12,400 households. CHARLS applied generalized multistage probability sampling strategy and probability-proportional-to-size (PPS) sampling technique. Four stages of sampling procedures (county-level sampling, neighborhood-level sampling, household-level sampling, and respondent-level sampling) were used to obtain a nationally representative sample [13]. In the sampling stage at the county-level, based on the population of each district and county in 2009 and using the region, urban and rural areas and GDP as hierarchical indications, 150 counties were randomly selected from 30 provincial administrative units (excluding Tibet Autonomous Region, Taiwan Province, Hong Kong and Macao Special Administrative Regions) in China according to the PPS method. In the sampling stage at the village level, based on the resident population of each village or community in 2009, three villages were randomly selected from each of the above 150 districts and counties, and finally 450 villages were obtained according to the PPS method. CHARLS performed the above sampling process in Stata software environment, and did not allow change of samples. To avoid the deviation of population information, the resident population data of 450 village units in 2009 were compared with those in 2007. For villages where the difference in population data over two years exceeded a certain limit, verification was obtained from the Bureau of statistics. Furthermore, for the selected villages, the quality of the sampling was guaranteed through the document issued by the Centers for Disease Control and Prevention (CDC) to the whole country for verification. The final sample included 450 administrative villages and neighborhoods in 150 counties, comprising more than 19,000 individual participants by 2018.

**Fig. 1:**
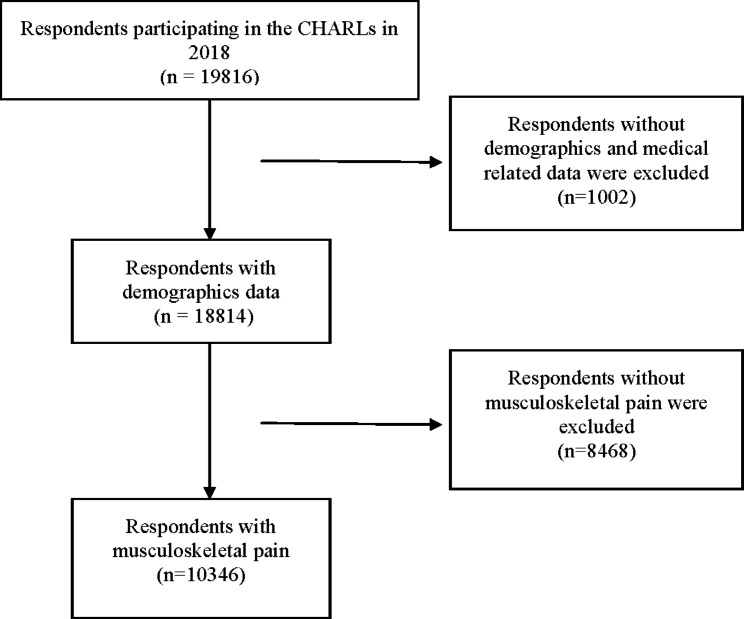
Data screening process for CHARLS data in 2018

The latest available CHARLS data in 2018 was selected to analyze the treatment preferences for musculoskeletal pain. The inclusion criteria for the present study were: (1) individuals aged at least 45 years old in CHARLS 2018; (2) and having data regarding musculoskeletal pain. Exclusion criteria were: (1) missing data of demographics and medical information; (2) persons aged less than 45 years old; (3) missing data of musculoskeletal pain in CHARLS 2018; (4) persons without musculoskeletal pain. After data screening, 1002 respondents were excluded for missing data, 18,814 respondents met the research requirements, of which 10,346 respondents met the requirements for musculoskeletal pain research (Fig. 1).

### Data collection and preprocessing

Information collected during the household interview included demographic characteristics (gender, age, residential address, marital status, employment), socioeconomic status (education, insurance status), health-related behavior (such as smoking, alcohol consumption).

When collecting information related to musculoskeletal pain, participants were first interviewed on whether they were “troubled” with any physical pain. If the answer was “a little”, “somewhat”, “quite a bit” or “very”, then they were asked to list all body parts that currently felt pain. Following this, participants were interviewed on whether they ever took any measures to reduce the pain, including Chinese traditional medicine, modern medicine, acupuncture treatment, and professional massage therapy. These answers were collected.

The age of subjects was categorized into 4 groups (45–54 years, 55–64 years, 65–74 years, and ≥ 75 years), as well as education level: no formal education, elementary school, middle/high school, and college degree or higher. All participants were classified as an urban or rural resident. The insurance situation of subjects was divided into 4 categories: no insurance, basic medical insurance, commercial insurance, and composite insurance (owning both basic medical insurance and commercial insurance). We divided the health-related behavior of respondents into three categories: “presently smoking/drinking” represented “yes” for smoking/drinking status, “previous smoking/drinking and quit now” represented “abstained from smoking/drinking”, and “never smoked/drank” represented “no” for smoking/drinking status. Smoking was defined as still smoking now, and drinking was defined as more than once a month in the past year. Musculoskeletal pain sites were categorized into 4 groups: neck pain, upper limb pain (including shoulder, arm, wrist and fingers), lower limb pain (including leg, knees, ankle and toes), and lower back pain (including back and waist). Some pain sites were excluded, such as the head, chest, and stomach. The number of pain sites were calculated based on the respondents’ answers regarding pain sites. The medical measures included Chinese traditional medicine, Western modern medicine, Acupuncture treatment, and Professional massage therapy. Other treatments were excluded due to the lack of a uniform description of answers. Stata 16.0 (StataCorp LP, USA) was used for data cleaning and processing.

### Machine-learning model and feature importance

Extreme gradient boosting (XGBoost) is an optimized algorithm for classifier based on the ensemble of weak learners. The XGBoost model was utilized in this study to analyze relevant factors by providing feature importance scores for each input data feature, aiding in the identification of the most significant features in the model. The contribution of each variable was evaluated using SHAP (Shaley values), which provided an explanation for the XGBoost model. In this process, the analysis and visualization were conducted using Python vision (3.8.3) [14, 15].

### Statistical analysis

To explore the effect factors of preference for musculoskeletal pain treatment, we selected the CHARLS data of 2018 (the latest data available). We performed univariate and multivariate analysis of treatment choice in different subgroups, using chi-square test and logistic regression. We used t-test to perform univariate analysis of treatment choice based on age. A P value of < 0.05 was considered statistically significant. All analysis and calculations were performed with R version 4.2.1.

## Results

### Demographics data

Among 19,816 respondents included in our analysis, 18,814 respondents met the research requirements (Demographics information shown in Supplementary Table 1). Among 18,814 respondents, 10,346 respondents suffered from musculoskeletal pain. As shown in Table 1, the majority of respondents were female, aged between 45 and 74, lived in rural areas, had no formal education, and were married. The coverage rate of resident insurance was above 90%. Most of the respondents did not smoke or drink, and were employed.

**Table 1:** Characteristics of respondents with musculoskeletal pain in CHARLS in 2018

Demographics	Sample(n = 10,346)
Gender, n (%)	
Male	4143 (40.04)
Female	6203 (59.96)
Age, years, mean ± SD	62.50 ± 10.02
Age, group, years, n (%)	
45–54	2697 (26.07)
55–64	3404 (32.90)
65–74	2885 (27.89)
≥ 75	1360 (13.15)
Residence, n (%)	
Rural	7990 (77.23)
Urban	2356 (22.77)
Education level, n (%)	
No formal education	5046 (48.77)
Elementary school	2259 (21.83)
Middle/high school	2713 (26.22)
College degree or higher	328 (3.17)
Marriage, n (%)	
Yes	8666 (83.76)
No	1680 (16.24)
Insurance status, n (%)	
No insurance	279 (2.70)
Basic medical insurance	9541 (92.22)
Commercial insurance	90 (0.87)
Composite insurance	436 (4.21)
Smoking status, n (%)	
Yes	2514 (24.30)
Abstinence	1344 (12.99)
No	6488 (62.71)
Drinking status, n (%)	
Yes	2351 (22.72)
Abstinence	373 (3.61)
No	7622(73.67)
Working status, n (%)	
Employed	6516 (62.98)
Unemployed	3830 (37.02)

CHARLS, China Health and Retirement Longitudinal Study

### Individual factors and preference

**Fig. 2:**
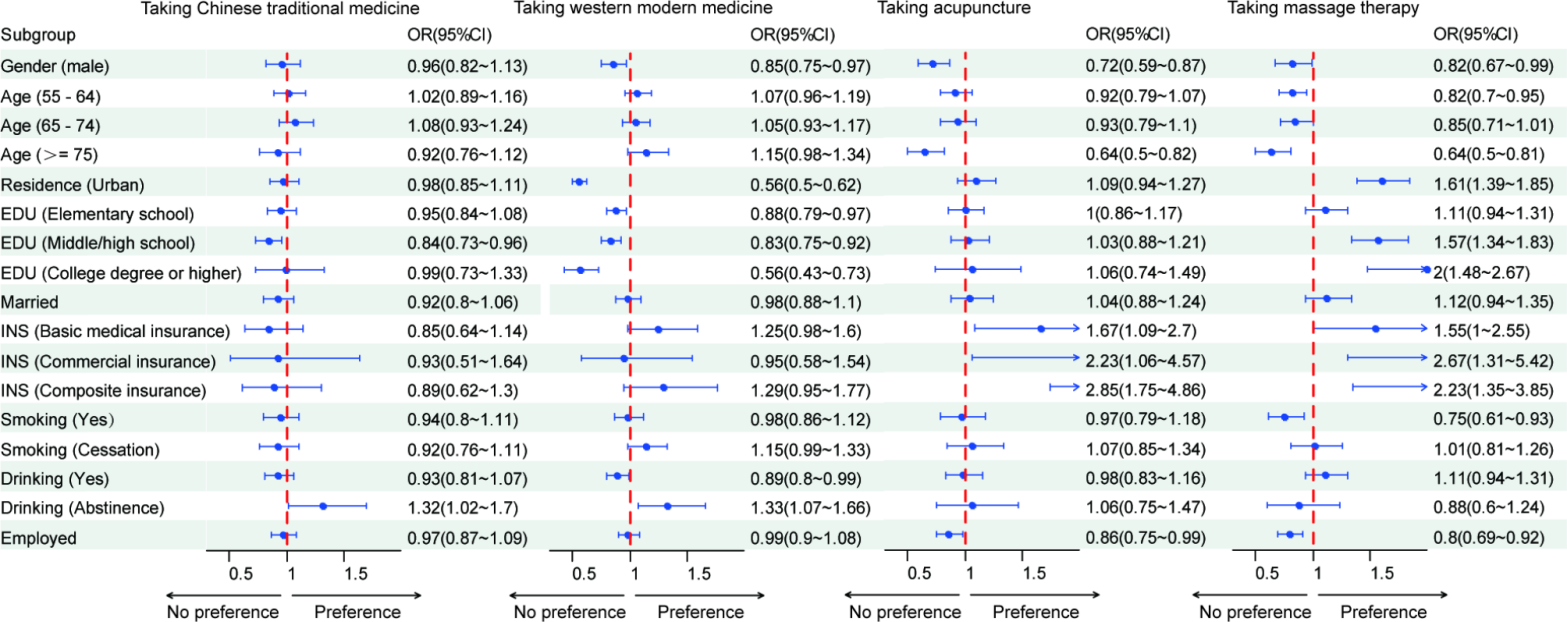
The multivariate analysis of treatment choice in different subgroups. The midpoint on the right side of the dotted red line indicates that this subgroup is more likely to choose this type of treatment, and the midpoint on the left is the opposite. EDU, education; OR, odds ratio

**Fig. 3:**
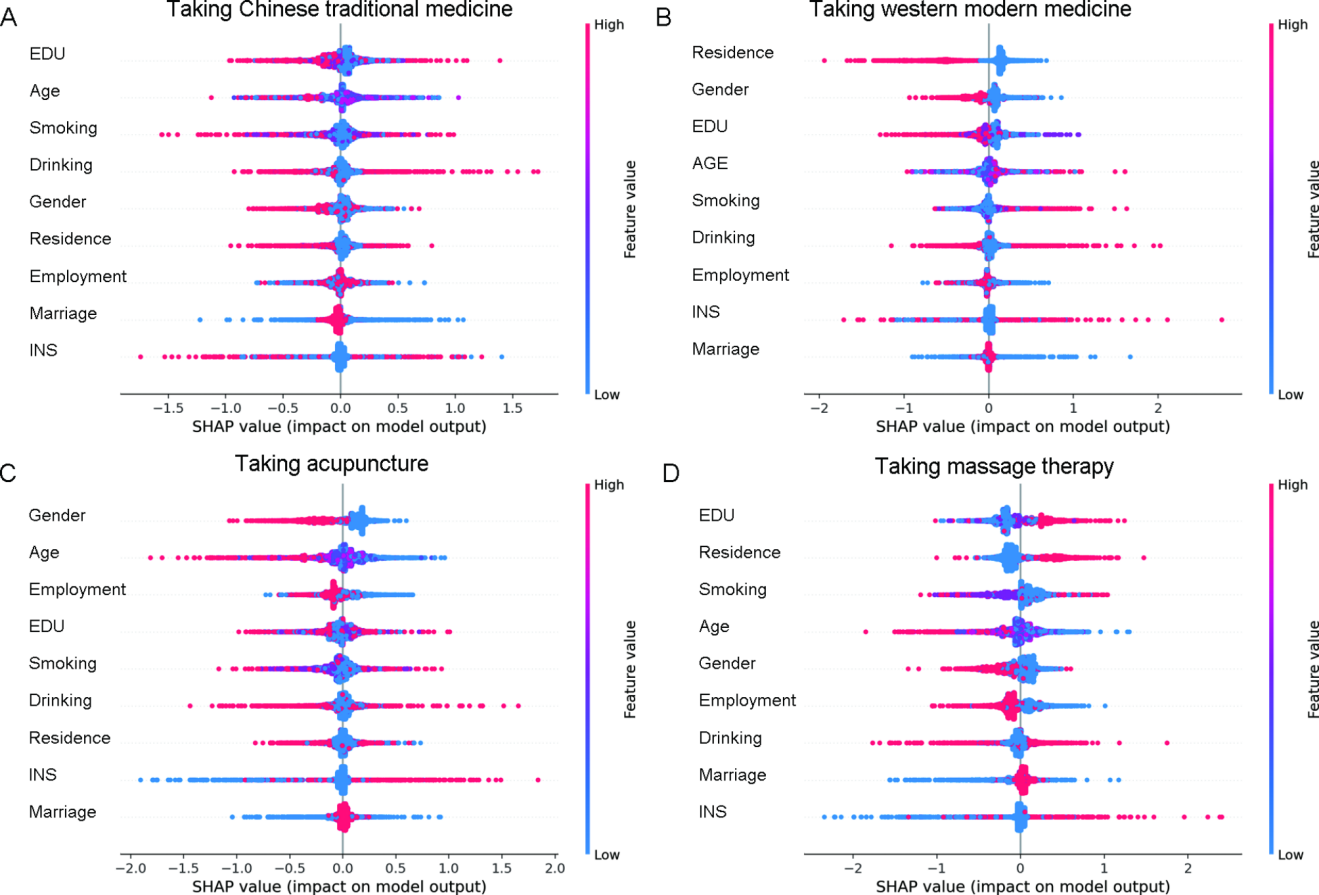
The rank of the importance of factors that influence the treatment preference for musculoskeletal pain: (**A**) factors that influence taking Chinese traditional medicine, (**B**) factors that influence taking modern medicine, (**C**) factors that influence taking acupuncture, (**D**) factors that influence taking massage therapy. EDU, education; INS, insurance

The results of univariate analysis on respondents’ preference in pain management are shown in Table 2. Variations in treatment preference of respondents with musculoskeletal pain were mainly related to gender, age, residence, education level, insurance status, and smoking and drinking. The multivariate analysis results were shown in Fig. 2 (Further details can be found in Supplementary Table 2). Chinese traditional medicine was less preferred by respondents with middle/high school education level (P < 0.05), and more preferred by those who were abstained from alcohol (P < 0.05). Modern medicine was less preferred by male respondents, those who lived in urban areas, and those with high education level (P < 0.05), and more preferred by those who were abstained from alcohol (P < 0.05). Acupuncture was less preferred by male respondents, those aged over 75, and those were employed (P < 0.05), and more preferred by those who had basic medical insurance or commercial insurance (P < 0.05). Massage therapy was less preferred by male respondents, those aged over 45, those who were smoking, and those who were employed (P < 0.05), and more preferred by those who lived in an urban area, those with middle school or higher education level, and those with basic medical insurance or commercial insurance (P < 0.05). The rank of the importance of these influencing factors were shown in Fig. 3. We utilized the SHAP explainer to calculate feature importance. The following features had a significant impact on the final prediction of the model: education, age, smoking, residence, gender, employment. The density scatter plot displayed all the samples with the ranking of features based on the sum of the average absolute values of SHAP. These results indicate the choice of medical care for musculoskeletal pain is mainly influenced by the patient’s gender, age, education level, and residential area.

**Table 2:** Univariate analysis of residents’ preference in pain management (n = 10,346)

Variables	Taking Chinese traditional medicine		Univariate analysis		Taking Western modern medicine		Univariate analysis		Taking Acupuncture		Univariate analysis		Taking Massage therapy		Univariate analysis	
	Yes, n	No, n	t/χ^2^	P	Yes, n	No, n	t/χ^2^	P	Yes, n	No, n	t/χ^2^	P	Yes, n	No, n	t/χ^2^	P
Gender			7.31	**0.01**			24.61	**< 0.01**			30.06	**< 0.01**			13.03	**< 0.01**
Male	743	3400			1812	2331			430	3713			456	3687		
Female	1245	4958			3021	3182			870	5333			831	5372		
Age, years			0.69	0.49			3.30	**< 0.01**			3.94	**< 0.01**			5.35	**< 0.01**
Mean ± SD	62.64 ± 9.84	62.47 ± 10.06			62.85 ± 10.07	62.20 ± 9.96			61.48 ± 9.52	62.65 ± 10.08			61.11 ± 9.64	62.70 ± 10.05		
Age			4.77	0.19			13.39	**< 0.01**			15.79	**< 0.01**			26.03	**< 0.01**
45–54	498	2199			1192	1505			370	2327			399	2298		
55–64	641	2763			1589	1815			433	2971			421	2983		
65–74	593	2292			1373	1512			369	2516			338	2547		
≥75	256	1104			679	681			128	1232			129	1231		
Residence			0.99	0.32			187.71	**< 0.01**			7.21	**0.01**			145.69	**< 0.01**
Rural	1552	6438			4024	3966			966	7024			824	7166		
Urban	436	1920			809	1547			334	2022			463	1893		
Education level			13.01	**0.01**			120.40	**< 0.01**			1.50	0.68			122.05	**< 0.01**
No formal education	1030	4016			2585	2461			624	4422			499	4547		
Elementary school	433	1826			1034	1225			277	1982			254	2005		
Middle/high school	462	2251			1123	1590			353	2360			454	2259		
College degree or higher	63	265			91	237			46	282			80	248		
Marriage			3.64	0.06			5.58	**0.02**			0.66	0.42			5.11	**0.02**
Yes	1637	7029			4004	4662			1099	7567			1106	7560		
No	351	1329			829	851			201	1479			181	1499		
Insurance status			1.91	0.59			5.02	0.17			29.26	**< 0.01**			35.15	**< 0.01**
No insurance	62	217			123	156			21	258			20	259		
Basic medical insurance	1823	7718			4486	5055			1178	8363			1162	8379		
Commercial insurance	19	71			36	54			14	76			17	73		
Composite insurance	84	352			188	248			87	349			88	348		
Smoking status			6.22	0.05			14.03	**< 0.01**			18.47	**< 0.01**			25.19	**< 0.01**
Yes	452	2062			1093	1421			263	2251			242	2272		
Cessation	241	1103			638	706			153	1191			168	1176		
No	1295	5193			3102	3386			884	5604			877	5611		
Drinking status			9.81	**0.01**			28.99	**< 0.01**			7.18	**0.03**			2.56	0.28
Yes	407	1944			991	1360			259	2092			287	2064		
Abstinence	86	287			197	176			44	329			37	336		
No	1495	6127			3645	3977			997	6625			963	6659		
Working status			1.18	0.28			0.61	0.44			3.57	0.06			18.42	**< 0.01**
Employed	1231	5285			3063	3453			788	5728			741	5775		
Unemployed	757	3073			1770	2060			512	3318			546	3284		

SD, Standard Deviation

### Pain sites and preference

**Fig. 4:**
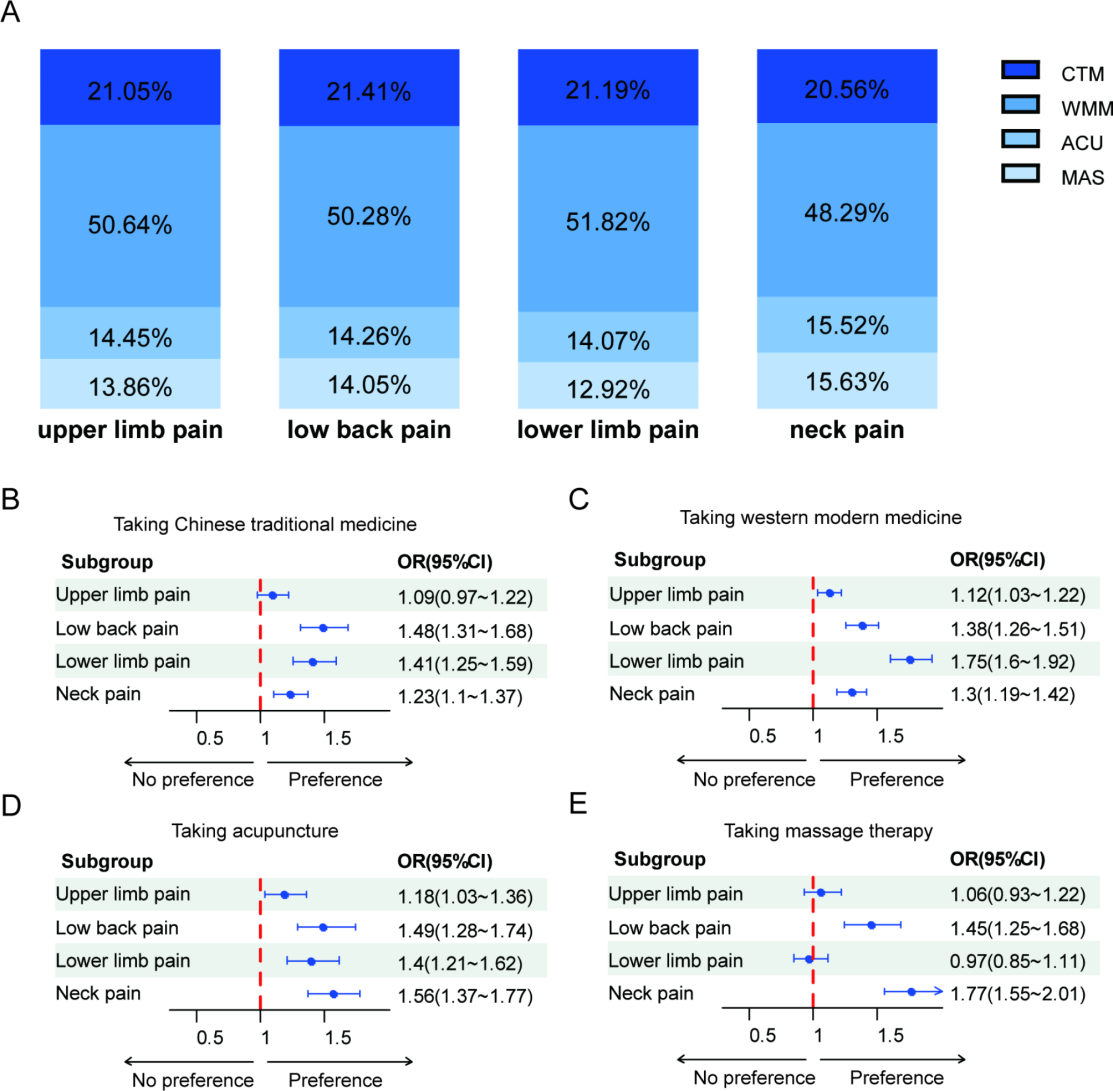
The treatment preference for different pain sites: (**A**) the proportion of treatment options for different pain sites. (**B**) The preference of taking Chinese traditional medicine for different pain sites. (**C**) The preference of taking modern medicine for different pain sites. (**D**) The preference of taking acupuncture for different pain sites. (**E**) The preference of taking massage therapy for different pain sites. CTM, Chinese traditional medicine; WMM, western modern medicine; ACU, acupuncture; MAS, massage

**Fig. 5:**
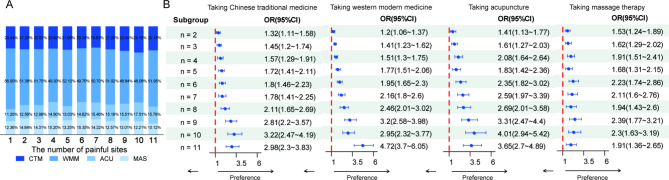
The treatment preference by the number of pain sites. (**A**) The proportion of treatment options by the number of pain sites. (**B**) The preference of different treatments by the number of pain sites. CTM, Chinese traditional medicine; WMM, western modern medicine; ACU, acupuncture; MAS, massage

The results of univariate analysis on the relationship between pain sites and treatment preference were shown in Table 3. The location of pain sites and the number of pain sites both influenced the preference for musculoskeletal pain treatment (P < 0.05). The treatment preferences for different musculoskeletal pain sites in 10,346 respondents were shown in Fig. 4 (Further details can be found in Supplementary Table 3). The location of the pain site did not influence the percentage of treatment choice, with all sites showing approximately 50% respondents taking modern medicine, 20% taking Chinese traditional medicine, and 15% taking acupuncture or massage therapy. All pain sites showed a positive impact on the respondents having a treatment preference rather than having no preference for the category of treatment. Compared with upper or lower limb pain, neck pain and lower back pain were more likely to have preferences for massage therapy (P < 0.05). A greater number of pain sites made it more likely for respondents to seek medical care for musculoskeletal pain (P < 0.05) (Fig. 5).

**Table 3:** Univariate analysis of residents’ preference in pain management (n = 10,346)

Variables	Taking Chinese traditional medicine		Univariate analysis		Taking Western modern medicine		Univariate analysis		Taking Acupuncture		Univariate analysis		Taking Massage therapy		Univariate analysis	
	Yes, n	No, n	t/χ^2^	P	Yes, n	No, n	t/χ^2^	P	Yes, n	No, n	t/χ^2^	P	Yes, n	No, n	t/χ^2^	P
Neck pain			44.53	**< 0.01**			102.14	**< 0.01**			104.12	**< 0.01**			116.09	**< 0.01**
Yes	788	2657			1851	1594			595	2850			599	2846		
No	1200	5701			2982	3919			705	6196			688	6213		
Upper limb pain			17.93	**< 0.01**			44.91	**< 0.01**			36.21	**< 0.01**			17.64	**< 0.01**
Yes	1361	5299			3274	3386			934	5726			896	5764		
No	627	3059			1559	2127			366	3320			391	3295		
Low back pain			55.56	**< 0.01**			72.23	**< 0.01**			50.48	**< 0.01**			44.88	**< 0.01**
Yes	1588	5988			3730	3846			1058	6518			1042	6534		
No	400	2370			1103	1667			242	2528			245	2525		
Lower limb pain			43.75	**< 0.01**			186.49	**< 0.01**			37.94	**< 0.01**			1.53	0.22
Yes	1550	5897			3790	3657			1029	6418			945	6502		
No	438	2461			1043	1856			271	2628			342	2557		
The number of painful sites, n			174.47	**< 0.01**			369.71	**< 0.01**			186.94	**< 0.01**			74.34	**< 0.01**
1	281	1863			770	1374			154	1990			170	1974		
2	300	1505			727	1078			178	1627			210	1595		
3	252	1156			622	786			156	1252			172	1236		
4	206	870			493	583			149	927			152	924		
5	194	747			468	473			117	824			119	822		
6	167	614			408	373			120	661			126	655		
7	127	472			328	271			100	499			92	507		
8	115	361			276	200			82	394			68	408		
9	127	300			274	153			87	340			73	354		
10	105	216			200	121			76	245			53	268		
11	114	254			267	101			81	287			52	317		

SD, Standard Deviation

## Discussion

Our analysis based on data from the CHARLS national population survey showed that treatment preferences for musculoskeletal pain in the Chinese population in 2018 was influenced by a variety of factors. Both respondent-related individual factors and factors relating to the presentation of musculoskeletal pain both led to statistically significant differences in treatment preference. Gaining a better understanding of these influencing factors are critical to improving the clinical management of musculoskeletal pain on a national level, and reducing the associated economic burden.

### Individual factors influencing treatment preferences for musculoskeletal pain

Pain is an inherently subjective and multidimensional experience comprising sensory, emotional, and cognitive components [16]. Genetic predisposition, gender, and mental processes such as feelings and beliefs surrounding pain contribute significantly to the interpretation of pain by individuals [17, 18]. In our study, a statistically significant result indicated that respondents with high education level had low preference for both Chinese traditional medicine and modern medicine. Interestingly, this coincides with numerous reports demonstrating worse outcomes in orthopedic patients with lower education levels [19], who have been associated with higher pain scores, decreased range of motion, and worse functional outcomes [20, 21]. The association between education level and experience of musculoskeletal pain is an interesting one that warrants further investigation.

There are multiple dimensions through which gender can influence the pain experience [22]. About 50% of chronic pain are more prevalent in women, while 20% are more common in men, such as migraine, musculoskeletal pain, and neuropathic pain. Gender differences also influence acute pain sensitivity [23]. For instance, physiological mechanisms underlying pain have sex-specific involvement of different genes and proteins, in addition to sex-specific interactions between hormones and the immune system that influence the transmission of pain signals. Testosterone is a sex hormone that is known to influence responses to suprathreshold, tonic stimuli, and pain tolerance [24]. For some conditions, women’s pain scores were reported to be more than 20% higher than men’s [25], which might be associated with sex-specific differences in pain sensitivity, tolerance, and willingness to report pain [26]. Interesting observations from a meta-analysis suggested that participants who considered themselves more “masculine” had higher pain thresholds, manifested by decreased pain sensitivity and increased pain tolerance [27]. This study similarly reported a statistically significant result that men preferred to not seek medical measures to relieve musculoskeletal pain.

Acupuncture is a complementary treatment modality derived from traditional Chinese medicine. During acupuncture, filiform needles are inserted into certain points on the body and stimulated with manual manipulation (twisting, pulling, and pushing), heat, or electrical pulses [28]. Therapeutic massage therapy is another complementary treatment modality that applies physical force to muscles, tendons, and connective tissues to promote muscule relaxation, reduce tension, relieve pain, and improve circulation [28]. Both therapies are recommended by clinical practice to treat musculoskeletal pain in grade C evidence [29]. However, there is a positive correlation between age and increased incidence of cancer, osteoarthritis, spinal diseases, surgical injuries, and other diseases which can directly lead to musculoskeletal pain [30]. To treat musculoskeletal pain caused by these diseases of aging, orthopedic surgeons have been suggested to choose therapies with higher grade evidence confirming their effects rather than acupuncture or massage therapy [31], which might also influence treatment preferences in aged patients.

Lifestyle factors involving intensive physical work, such as for agricultural activities are more common in rural than urban areas [30]. At the same time, rural residents are more likely to engage in manual labor, and are often associated with low education level and low coverage of health insurance. These factors have been reported to be associated with higher prevalence of pain and greater pain scores [30], which may in turn lead to differences in treatment preferences for musculoskeletal pain.

### Current status of musculoskeletal pain treatment in the chinese population

The results of our analysis showed that about 50% of respondents chose modern medicine to relieve musculoskeletal pain, followed by 20% on Chinese traditional medicine. A possible reason for this result is that pharmacological treatment as part of modern medicine is often preferred by clinicians for acute or chronic pain management as it is considered a simple and effective basic treatment strategy [32, 33]. Modern medicine and traditional Chinese medicine practices co-exist at all levels within the Chinese healthcare system. It should be noted that a portion of the Chinese population of clinicians and patients have a strong preference in choosing traditional Chinese medicine for musculoskeletal pain [12, 34]. Nevertheless, the majority of respondents in our study chose modern medicine regardless of the location of pain. According to a survey conducted in approximately 1000 orthopedic surgeons in China, about 50% applied modern medicine for musculoskeletal pain by referring to treatment guidelines, coinciding with the proportion of respondents choosing modern medicine in our study. In addition, another factor contributing to this result might be that the CHARLS survey population was predominantly older people with low education levels living in rural China, who are more likely to be associated with higher pain scores and follow the standard medical care prescribed by clinicians [20, 21, 30].

Unlike for other treatment modalities, respondents who preferred massage therapy appeared to be predominantly affected by neck and lower back pain. Some reviews have shown low strength findings suggesting potential benefits of massaging in pain relief, including for the shoulder, neck and low back [35], but these were not rated as moderate or high strength evidence. As major health problems that represent the leading causes of years lived with disability and significant sources of societal burden, long-term effective interventions are still lacking and call for further research [36].

### Study strengths and limitations

Based on CHARLS data, this study conducted an in-depth analysis of the Chinese elderly population on their treatment preferences for musculoskeletal pain. Our study summarized the characteristics of 18,814 respondents, among which 10,346 respondents suffering from musculoskeletal pain were selected for a comprehensive investigation of their pain data and treatment related information, including subgroup analysis on age, gender, socioeconomic status, and health-related behavior. This is the first study to have performed comprehensive analysis on this large population of patients on the nationwide Chinese population to better understand the status of musculoskeletal pain management and treatment preferences by patients within the country. Our study comprehensively analyzed the outcome of treatment choice of respondents with different individual characteristics and pain sites, through which we summarized the factors influencing treatment preferences and ranked the importance of these factors by the random forest method.

The results of our study should be interpreted with consideration given to a number of limitations. Firstly, there was a certain number of missing values in the CHARLS 2018 data, which may have resulted in some level of selection bias. Secondly, this study lacked a specific scale for the collection of pain data, which coupled with the high subjectivity of pain experience might have resulted in some inconsistency in the reporting of pain data from respondents. Thirdly, in the questionnaire, some data related to musculoskeletal pain were not independent of each other, such as different pain sites or treatment choices exist simultaneously, which may have resulted in bias during related data processing. Finally, this study categorized the musculoskeletal pain sites into neck, lower back, upper and lower limb pain, but musculoskeletal pain often involves more specific sites such as shoulders and knees. Further research is needed to better delineate the associations between specific common pain sites and treatment preferences. Nevertheless, our study provides new insight and fills a critical gap in information on treatment choices for Chinese patients with musculoskeletal pain. This new information on patient treatment preferences may affect patient adherence during long-term treatment and be useful in guiding clinical decision making in the community for different painful sites or different population.

## Conclusions

Pharmacological therapies as part of modern medicine played an important role in the management of Chinese patients with musculoskeletal pain and was the preferred treatment modality, while massage therapy was preferred by patients with neck and lower back pain. Gender, age, education level, and area of residence had potential effects on treatment preferences for musculoskeletal pain in the Chinese population, while different pain sites had little influence. A greater number of pain sites was associated with a higher likelihood for people to seek medical care for musculoskeletal pain.

## Electronic supplementary material

Below is the link to the electronic supplementary material.

Supplementary Material 1

## Data Availability

The datasets used and/or analyzed during the current study are available from the corresponding author on reasonable request. All data was from http://charls.pku.edu.cn/.
