# Supplementary material for: Preference of musculoskeletal pain treatment in middle-aged and elderly chinese people: a machine learning analysis of the China health and retirement longitudinal study
Source: BMC Musculoskelet Disord. 2023 Jun 29;24:528. doi: 10.1186/s12891-023-06665-7 (PMC10308742; doi:10.1186/s12891-023-06665-7)
Supplement: Supplementary file 1 — Supplementary Material 1 [file 12891_2023_6665_MOESM1_ESM.docx]

**Supplementary Table 1 Characteristics of respondents in CHARLS in 2018**

| **Demographics** | **Sample（n=18814）** |
| --- | --- |
| Gender, n (%) |  |
| Male | 8981 (47.74) |
| Female | 9833 (52.26) |
| Age, years, mean ± SD | 62.14±10.08 |
| Age, group, years, n (%) |  |
| 45-54 | 5175 (27.51) |
| 55-64 | 6252 (33.23) |
| 65-74 | 5003 (26.59) |
| ≥75 | 2384 (12.67) |
| Residence, n (%) |  |
| Rural | 14030 (74.57) |
| Urban | 4784 (25.43) |
| Education level, n (%) |  |
| No formal education | 8161 (43.38) |
| Elementary school | 4162 (22.12) |
| Middle/high school | 5670 (30.14) |
| College degree or higher | 821 (4.36) |
| Marriage, n (%) |  |
| Yes | 16016 (85.13) |
| No | 2798(14.87) |
| Insurance status, n (%) |  |
| No insurance | 546 (2.90) |
| Basic medical insurance | 17241 (91.64) |
| Commercial insurance | 164 (0.87) |
| Composite insurance | 863 (4.59) |
| Smoking status, n (%) |  |
| Yes | 5086 (27.03) |
| Abstinence | 2745 (14.59) |
| No | 10983 (58.38) |
| Drinking status, n (%) |  |
| Yes | 4937 (26.24) |
| Abstinence | 702 (3.73) |
| No | 13175 (70.03) |
| Working status, n (%) |  |
| Employed | 11982 (63.69) |
| Unemployed | 6832 (36.31) |

CHARLS, China Health and Retirement Longitudinal Study

**Supplementary Table 2 Multivariate analysis of residents’ preference in pain management (n=10346)**

| **Variables** | **Taking Chinese traditional medicine** | | Multivariate analysis | | **Taking Western modern medicine** | | Multivariate analysis | | **Taking Acupuncture** | | Multivariate analysis | | **Taking Massage therapy** | | Multivariate analysis | |
| --- | --- | --- | --- | --- | --- | --- | --- | --- | --- | --- | --- | --- | --- | --- | --- | --- |
|  | Yes, n | No, n | OR | P | Yes, n | No, n | OR | P | Yes, n | No, n | OR | P | Yes, n | No, n | OR | P |
| Gender |  |  |  |  |  |  |  |  |  |  |  |  |  |  |  |  |
| Male | 743 | 3400 | 0.96 | 0.62 | 1812 | 2331 | 0.85 | **0.02** | 430 | 3713 | 0.72 | **<0.01** | 456 | 3687 | 0.82 | **0.05** |
| Female | 1245 | 4958 | ref | ref | 3021 | 3182 | ref | ref | 870 | 5333 | ref | ref | 831 | 5372 | ref | ref |
| Age |  |  |  |  |  |  |  |  |  |  |  |  |  |  |  |  |
| 45-54 | 498 | 2199 | ref | ref | 1192 | 1505 | ref | ref | 370 | 2327 | ref | ref | 399 | 2298 | ref | ref |
| 55-64 | 641 | 2763 | 1.02 | 0.79 | 1589 | 1815 | 1.07 | 0.22 | 433 | 2971 | 0.92 | 0.27 | 421 | 2983 | 0.82 | **0.01** |
| 65-74 | 593 | 2292 | 1.08 | 0.31 | 1373 | 1512 | 1.05 | 0.43 | 369 | 2516 | 0.93 | 0.40 | 338 | 2547 | 0.85 | 0.06 |
| ≥75 | 256 | 1104 | 0.92 | 0.41 | 679 | 681 | 1.15 | 0.08 | 128 | 1232 | 0.64 | **<0.01** | 129 | 1231 | 0.64 | **<0.01** |
| Residence |  |  |  |  |  |  |  |  |  |  |  |  |  |  |  |  |
| Rural | 1552 | 6438 | ref | ref | 4024 | 3966 | ref | ref | 966 | 7024 | ref | ref | 824 | 7166 | ref | ref |
| Urban | 436 | 1920 | 0.98 | 0.71 | 809 | 1547 | 0.56 | **<0.01** | 334 | 2022 | 1.09 | 0.25 | 463 | 1893 | 1.61 | **<0.01** |
| Education level |  |  |  |  |  |  |  |  |  |  |  |  |  |  |  |  |
| No formal education | 1030 | 4016 | ref | ref | 2585 | 2461 | ref | ref | 624 | 4422 | ref | ref | 499 | 4547 | ref | ref |
| Elementary school | 433 | 1826 | 0.95 | 0.46 | 1034 | 1225 | 0.88 | **0.02** | 277 | 1982 | 1.00 | 0.96 | 254 | 2005 | 1.11 | 0.21 |
| Middle/high school | 462 | 2251 | 0.84 | **0.01** | 1123 | 1590 | 0.83 | **<0.01** | 353 | 2360 | 1.03 | 0.70 | 454 | 2259 | 1.57 | **<0.01** |
| College degree or higher | 63 | 265 | 0.99 | 0.95 | 91 | 237 | 0.56 | **<0.01** | 46 | 282 | 1.06 | 0.74 | 80 | 248 | 2.00 | **<0.01** |
| Marriage |  |  |  |  |  |  |  |  |  |  |  |  |  |  |  |  |
| Yes | 1637 | 7029 | 0.92 | 0.25 | 4004 | 4662 | 0.98 | 0.76 | 1099 | 7567 | 1.04 | 0.63 | 1106 | 7560 | 1.12 | 0.22 |
| No | 351 | 1329 | ref | ref | 829 | 851 | ref | ref | 201 | 1479 | ref | ref | 181 | 1499 | ref | ref |
| Insurance status |  |  |  |  |  |  |  |  |  |  |  |  |  |  |  |  |
| No insurance | 62 | 217 | ref | ref | 123 | 156 | ref | ref | 21 | 258 | ref | ref | 20 | 259 | ref | ref |
| Basic medical insurance | 1823 | 7718 | 0.85 | 0.27 | 4486 | 5055 | 1.25 | 0.07 | 1178 | 8363 | 1.67 | **0.03** | 1162 | 8379 | 1.55 | 0.06 |
| Commercial insurance | 19 | 71 | 0.93 | 0.80 | 36 | 54 | 0.95 | 0.83 | 14 | 76 | 2.23 | **0.03** | 17 | 73 | 2.67 | **0.01** |
| Composite insurance | 84 | 352 | 0.89 | 0.56 | 188 | 248 | 1.29 | 0.11 | 87 | 349 | 2.85 | **<0.01** | 88 | 348 | 2.23 | **<0.01** |
| Smoking status |  |  |  |  |  |  |  |  |  |  |  |  |  |  |  |  |
| Yes | 452 | 2062 | 0.94 | 0.49 | 1093 | 1421 | 0.98 | 0.79 | 263 | 2251 | 0.97 | 0.75 | 242 | 2272 | 0.75 | **0.01** |
| Cessation | 241 | 1103 | 0.92 | 0.39 | 638 | 706 | 1.15 | 0.07 | 153 | 1191 | 1.07 | 0.58 | 168 | 1176 | 1.01 | 0.92 |
| No | 1295 | 5193 | ref | ref | 3102 | 3386 | ref | ref | 884 | 5604 | ref | ref | 877 | 5611 | ref | ref |
| Drinking status |  |  |  |  |  |  |  |  |  |  |  |  |  |  |  |  |
| Yes | 407 | 1944 | 0.93 | 0.30 | 991 | 1360 | 0.89 | **0.04** | 259 | 2092 | 0.98 | 0.81 | 287 | 2064 | 1.11 | 0.23 |
| Abstinence | 86 | 287 | 1.32 | **0.03** | 197 | 176 | 1.33 | **0.01** | 44 | 329 | 1.06 | 0.72 | 37 | 336 | 0.88 | 0.47 |
| No | 1495 | 6127 | ref | ref | 3645 | 3977 | ref | ref | 997 | 6625 | ref | ref | 963 | 6659 | ref | ref |
| Working status |  |  |  |  |  |  |  |  |  |  |  |  |  |  |  |  |
| Employed | 1231 | 5285 | 0.97 | 0.63 | 3063 | 3453 | 0.99 | 0.75 | 788 | 5728 | 0.86 | **0.03** | 741 | 5775 | 0.80 | **<0.01** |
| Unemployed | 757 | 3073 | ref | ref | 1770 | 2060 | ref | ref | 512 | 3318 | ref | ref | 546 | 3284 | ref | ref |

SD, Standard Deviation

**Supplementary Table 3 Multivariate analysis of residents’ preference in pain management (n=10346)**

| **Variables** | **Taking Chinese traditional medicine** | | Multivariate analysis | | **Taking Western modern medicine** | | Multivariate analysis | | **Taking Acupuncture** | | Multivariate analysis | | **Taking Massage therapy** | | Multivariate analysis | |
| --- | --- | --- | --- | --- | --- | --- | --- | --- | --- | --- | --- | --- | --- | --- | --- | --- |
|  | Yes, n | No, n | OR | P | Yes, n | No, n | OR | P | Yes, n | No, n | OR | P | Yes, n | No, n | OR | P |
| Neck pain |  |  |  |  |  |  |  |  |  |  |  |  |  |  |  |  |
| Yes | 788 | 2657 | 1.23 | **<0.01** | 1851 | 1594 | 1.30 | **<0.01** | 595 | 2850 | 1.56 | **<0.01** | 599 | 2846 | 1.77 | **<0.01** |
| No | 1200 | 5701 | ref | ref | 2982 | 3919 | ref | ref | 705 | 6196 | ref | ref | 688 | 6213 | ref | ref |
| Upper limb pain |  |  |  |  |  |  |  |  |  |  |  |  |  |  |  |  |
| Yes | 1361 | 5299 | 1.09 | 0.13 | 3274 | 3386 | 1.12 | **0.01** | 934 | 5726 | 1.18 | **0.02** | 896 | 5764 | 1.06 | 0.38 |
| No | 627 | 3059 | ref | ref | 1559 | 2127 | ref | ref | 366 | 3320 | ref | ref | 391 | 3295 | ref | ref |
| Low back pain |  |  |  |  |  |  |  |  |  |  |  |  |  |  |  |  |
| Yes | 1588 | 5988 | 1.48 | **<0.01** | 3730 | 3846 | 1.38 | **<0.01** | 1058 | 6518 | 1.49 | **<0.01** | 1042 | 6534 | 1.45 | **<0.01** |
| No | 400 | 2370 | ref | ref | 1103 | 1667 | ref | ref | 242 | 2528 | ref | ref | 245 | 2525 | ref | ref |
| Lower limb pain |  |  |  |  |  |  |  |  |  |  |  |  |  |  |  |  |
| Yes | 1550 | 5897 | 1.41 | **<0.01** | 3790 | 3657 | 1.75 | **<0.01** | 1029 | 6418 | 1.40 | **<0.01** | 945 | 6502 | 0.97 | 0.65 |
| No | 438 | 2461 | ref | ref | 1043 | 1856 | ref | ref | 271 | 2628 | ref | ref | 342 | 2557 | ref | ref |

SD, Standard Deviation
